# 拉曼光谱检测技术在早期肺癌诊断方面的研究进展

**DOI:** 10.3779/j.issn.1009-3419.2018.07.10

**Published:** 2018-07-20

**Authors:** 笑如 田, 毅 张

**Affiliations:** 100053 北京，首都医科大学宣武医院胸外科 Department of Thoracic Surgery, Xuanwu Hospital, Capital Medical University, Beijing 100053, China

**Keywords:** 肺肿瘤, 早期诊断, 拉曼光谱, Lung neoplasms, Early diagnosis, Raman spectroscopy

## Abstract

肺癌是目前世界范围内发病率和死亡率最高的恶性肿瘤，5年生存率仅18%，伴有远处转移的患者则为5%，而Ⅰ期非小细胞肺癌患者5年生存率最高达77.9%，因此肺癌的早诊早治是改善预后的关键。拉曼光谱技术作为一种非侵入性的检测技术，可实现对癌变组织与正常组织之间分子水平结构差异的检测，从而可用于肺癌的早期诊断。该文综述了拉曼光谱检测技术结合不同组织或体液样本在早期肺癌诊断方面的研究进展。

## Abstract (en)

Lung cancer (LC) is the most common cancer and the leading cause of cancer-related death worldwide. The 5-year survival rate for LC remains low at 18% and is 5% for patients with metastatic disease, while the 5-year overall survival rate of patients with stage Ⅰ NSCLC can reach 77.9%, hence early diagnosis and treatment of LC is the key to improve the prognosis. As a non-invasive detection technique, Raman spectroscopy can realize the non-destructive detection of the differences in molecular level structure between cancerous tissues and normal tissues, which can be used for the early diagnosis of lung cancer. The aim of this review is to summarize the progress of Raman spectroscopycombined with different tissue or body fluid samplesin the diagnosis of early LC.

肺癌是目前世界范围内发病率和死亡率最高的恶性肿瘤，全球年新发肺癌患者约182.5万人，死亡160万人^[1]^，而中国年肺癌新发及死亡人数则分别为约78.1万和62.6万^[2]^。数据显示肺癌患者5年生存率低至18%，伴有远处转移的患者则为5%^[3]^。究其原因，早期肺癌多无明显临床表现，多数患者发现肺癌时已至晚期，错失最佳治疗时机，预后不佳。有研究表明Ⅰ期非小细胞肺癌（non-small cell lung cancer, NSCLC）肺癌患者5年总生存期（overall survival, OS）能达到73.5%-77.9%，5年无病生存（disease-free survival, DFS）率为65.5%-72.7%^[4]^。因此，早期肺癌诊断对于提高肺癌早治率，降低患者死亡率非常重要。

目前临床常用早期肺癌诊断方法包括血肿瘤标志物、胸部X线片、胸部计算机断层扫描（computed tomography, CT）、纤维支气管镜、支气管超声引导下针吸活检术、经皮肺穿刺活检等，在早期肺癌诊断方面表现出了很好的临床效用，但或多或少存在敏感度差、准确率低、成本高、周期长、有创等问题。因此研究一种敏感度强、特异性高、无创、简单有效、成本低的诊断技术等具有重要临床意义。拉曼光谱检测技术作为一种非侵入性微量物质检测技术，具有灵敏度高、特异度好、快速有效等优点，在癌症诊断方面表现出了良好的临床应用前景。本文通过复习文献，对拉曼光谱检测技术在肺癌诊断方面的应用发展作一综述。

## 1 拉曼光谱检测技术的原理

拉曼光谱最早于1928年由印度物理学家Raman等^[5]^发现，是研究分子振动的一种光谱学方法，它可以实现分子化学物质的指纹识别，从而实现生物分子的无损检测。在癌症检测方面，拉曼光谱可以探测到与癌变相关的微小分子层次变化，如核质比增加、染色质无序、高代谢活性、脂质和蛋白质分子水平变化等，通过对比研究癌组织和正常组织的拉曼光谱，可发现其能反映组织病变信息的特征光谱，因此拉曼光谱技术可能实现对癌症的早期诊断，进而指导早期治疗。

然而，物质自发拉曼光谱信号极弱，且极易受荧光干扰，灵敏度低，应用范围受到极大限制。表面增强拉曼效应（surface enhanced raman spectroscopy, SERS）的出现解决了这个问题。SERS是通过吸附在金属纳米结构表面上的分子与金属表面发生的等离子共振相互作用而引起的拉曼散射强度增强的现象，是一种非常有效的拉曼信号检测技术。SERS技术核心在于增强基底，一般SERS增强因子的平均值约为10^6^，但是在纳米粒子的间隙位置和尖锐突起外表面，局部增强可达到10^14^。然而目前SERS基底的稳定性较差，而基底的细微差异就会使测量结果的变化很大^[6]^，目前仍无稳定性和重现性均较高的增强基底^[7]^，仍不适于实际样品中痕量目标物的定量分析。为此，专家们进行了大量探索研究，如开发不同增强基底^[6, 8-10]^、表面增强纳米修饰芯片^[11]^，开发磁辅助表面增强拉曼散射技术^[12]^、共焦显微拉曼技术^[13]^、表面增强相干反斯托克斯-拉曼散射技术^[14]^等。也有专家将拉曼光谱技术与内镜结合对病灶进行实时照射活检，也表现出不错的效果^[15, 16]^。已有大量研究表明拉曼光谱检测技术可实现对卵巢癌^[13]^、结直肠癌^[17]^、宫颈癌^[18]^、前列腺癌^[19]^等癌症的诊断。但是检测技术稳定性及重现性欠佳、底物预处理可能影响结果、检测品类单一等问题仍未得到合理解决^[7]^。且对于液体样品而言，检测灵敏度仍不够高，通常只能检测浓度较高的溶液。

液芯波导增强拉曼光谱检测技术（liquid core waveguide enhanced raman spectroscopy, LOWERS）是通过使激光在液体/波导管界面上形成内部全反射，使其通过样品的有效光程远大于波导管的几何长度，从而显著提高信号强度、检测灵敏度和探测极限。LOWERS是一种纯光学增强，能实现对液体样品中不同物质的同步痕量检测，虽灵敏性略低于应用最广的SERS，但其稳定性远高于SERS。以非结晶无定形含氟聚合物（Teflon AF2400）制成的波导管能使LOWERS达到10^3^量级的拉曼光谱信号放大，灵敏度和S/N也成比例增高^[20]^，尤其适用于水溶液特别是体液检测。但当波导管液体中因进样时随机出现的气泡使光路中断时，增强效果会受到显著影响，进而影响检测结果。为此，刘霁欣等^[21]^开发了负压式LOWERS，可将Teflon AF管中气泡经由透气的Teflon AF管壁抽出，避免其对LOWERS信号产生影响，该技术可对微升级液体实现100倍以上的稳定增敏。

## 2 拉曼光谱检测技术诊断肺癌

### 2.1 肺组织拉曼光谱检测技术诊断肺癌的研究进展

唐伟跃等^[22]^应用共焦显微拉曼光谱技术对肺癌及癌旁正常组织切片进行分析，得出两种组织的谱图在峰频移及峰强度方面表现出明显差异：①正常肺组织切片的1, 392/cm附近的C-H变形振动在癌变组织光谱中向高波数移动约6/cm; ②1, 523/cm脂类胡萝卜素的C=C振动谱带在癌变组织光谱中向高波数移动约3/cm; ③在正常组织中1, 610/cm处谱带的强度明显的强于1, 526/cm处谱带的强度，而在癌变组织中则正好相反。表明组织细胞在癌变过程中在构型、构象和数量上发生了变化，提示该方法在揭示恶性肿瘤的发生发展规律及其他良性疾病的基础研究中有着良好的前景。McGregor等^[16]^团队开发了快速实时内镜拉曼光谱检测系统和专用于肺部检测的内窥镜拉曼导管，能使检测时间缩短到1 s以内^[23]^，结合自体荧光支气管镜+白光支气管镜技术，对后两者筛查出的可疑区域进行拉曼光谱检测，研究共检测了80例病人的280例组织（包括72例高度异型性变或恶性病变组织及208例良性或正常组织），结果显示其对高度异型性变和恶性病变的检出具有高敏感度（90%）和很好的特异度（65%），检测快速准确。Weng等^[14]^采用相干反斯托克斯-拉曼散射技术结合深度学习算法研究肺癌的自动鉴别诊断，判别正常、小细胞肺癌、腺癌及鳞癌肺组织的准确度能达到89.2%。然而肺组织样本的获取创伤大、成本高，实时内镜拉曼光谱技术操作复杂且创伤大，技术尚不成熟，仍需要大量研究以挖掘其在诊断肺癌及癌前病变方面的潜力。

### 2.2 呼出气中挥发性有机物的拉曼光谱检测诊断肺癌的研究进展

人呼出气中有超过3, 000种挥发性有机物（volatile organic compounds, VOCS），可能有助于早期肺癌检测^[24]^。有研究^[25-27]^证明呼出气中多种VOCS可作为肺癌相关生物标志物，有助于肺癌诊断。Zhang等^[8]^采用基于树枝状银纳米晶体的表面增强拉曼光谱技术，利用“腔窝”效应延长气态分子在固体表面的反应时间，通过与树突状银纳米晶体上预接枝的拉曼活性探针分子对巯基苯胺的亲核加成反应，能敏感地捕获气态醛分子，并实现十亿分率水平的检测。该方法对肺癌生物标志物检测的敏感性不受湿度的影响，这使其在快速、简便、经济、无创地判别肺癌方面具有很大的潜力。该团队也研究了采用基于ZIF-8涂层的金超粒子自组装体的表面增强拉曼光谱技术捕获气态醛，检出限能达到100亿分率水平，显示了其在早期肺癌体外诊断方面的良好应用前景^[9]^。VOCS拉曼光谱检测无创、快速，在肺癌诊断方面表现出了良好潜力，但实际临床效用仍有较大争议，相关研究较少，但仍需进一步研究探讨。

### 2.3 唾液拉曼光谱检测技术诊断肺癌的研究进展

唾液含有大量与体征相关的蛋白质和分泌物质，显示出对多种疾病诊断的潜力^[28]^。李晓舟等^[29]^采用以银胶为基底的SERS结合倒置显微拉曼光谱仪检测肺癌患者和健康人唾液样本拉曼光谱，以主成分分析（principle components analysis, PCA）和线性判别分析对光谱数据降维和区分，结果显示从对照组到肺癌组蛋白质和核酸含量的整体下降，人肺癌肿瘤细胞的拉曼光谱也表明核酸和蛋白质相对正常人的下降，表明唾液在某种程度上可以反映组织的状况，判别肺癌的准确度、灵敏度和特异性分别能达到84%、94%和81%，揭示了唾液SERS用于诊断肺癌的潜力。但是SERS检测技术的稳定及重现性不佳的问题仍未解决。Liu等^[11]^采用基于表面增强纳米修饰芯片的便携式拉曼光谱系统检测分析肺癌患者和健康人唾液样本，通过对特征峰的研究分析发现肺癌患者和健康人唾液中蛋白质分子结构和核酸分子种类及含量上明显不同，建立的SVM模型和随机森林模型判别肺癌的灵敏度、特异性、准确度分别为100%、94.44%、98.3%和100%、88.8%、97.4%。唾液样本获取方便、完全无创，且在研究中表现出了高特异性、灵敏度及准确度，但是该检测系统依赖的芯片精度虽高但生产技术尚不成熟，不同批次芯片间的差异较大，稳定性及重现性差。且唾液样本易受痰液干扰，可能会使结果有所偏倚。

### 2.4 尿液拉曼光谱检测在肺癌诊断方面的研究进展

尿液中含有大约3, 000种蛋白质，是一种理想的蛋白质和肽类生物标记物研究样本^[30]^。Niu等^[31]^采用气相色谱/质谱法对尿液样本进行代谢组学分析，发现尿液中代谢产物共38种，其中有7种能有效指示肺癌，包括肌酐、肌醇、3-羟基丁酸、核苷酸、柠檬酸、十六酸、油酸，除了核苷酸在肺癌组下降外，其余6种物质在肺癌组水平均高于其他肺部疾病组。也有大量研究表明尿液中GM2AP（GM2-activator protein）^[32]^、血浆铜蓝蛋白^[33]^、肌酸核苷和N-乙酰神经氨酸^[34]^、修饰核苷^[35]^等与肺癌有密切关系，有助于早期肺癌诊断。尿液分析完全无创、取材方便，尿代谢物生物标志物作为无创临床筛查工具对肺癌的早期诊断具有潜在的实用价值^[36]^。金刚烷胺的乙酰化在肿瘤细胞中明显上调可用于癌症尤其是肺癌的检测，但由于其在尿液中浓度很低，一般方法难以检测，有研究^[37]^采用基于有环式糊精包绕的金属基底的SERS检测技术，可实现对肺癌患者尿液样本中乙酰金刚烷胺的检测，极限检测浓度能达到1 ng/mL，且具有成本低、快速的优点，有望成为临床早期肺癌的诊断手段。此外，腺苷可能是一种癌症相关生物标志物。人血清是一种含有蛋白质的高度复杂的基质，对腺苷的直接测定有严重的干扰作用，而尿液由盐和小分子组成，如尿素和核苷，适于测定腺苷。Yang等^[12]^通过合成由植酸及其盐编织并稳定化的Fe_3_O_4_/Au/Ag纳米复合材料，开发了磁辅助表面增强拉曼光谱技术，检测肺癌患者和健康人尿液样本中痕量水平的腺苷，能达到1×10^-10^ mol/L浓度的检测水平，具有灵敏度高、选择性好、预处理简单、检测速度快等优点，可以有效地指示肺癌。但是尿中尿素可造成对腺苷检测的严重干扰，虽然偶氮偶联能成功去除尿中尿素，但也会严重干扰腺苷的SERS信号。这些研究证实了尿液拉曼光谱检测诊断肺癌的优势及其可行性，具有良好的应用前景。但是尿液中肺癌相关生物标志物的种类及含量仍无国际公认标准，尿液背景物质对样本拉曼信号的干扰仍未得到有效解决，且相关检测技术不成熟，稳定性及重现性差的问题仍是限制其临床广泛应用的瓶颈。

### 2.5 其他样品拉曼光谱检测诊断肺癌的研究进展

Ye等^[38]^基于某些microRNA^[39]^可作为肺癌标志物的研究，开发了基于循环指数扩增反应（EXPAR）的SERS方法，可同时检测肺癌中的多种microRNA，并能从高度同源的microRNA中区分出目标microRNA，在临床早期肺癌诊断中有很大的应用潜力。Park等^[40]^利用SERS分别对NSCLC细胞和正常肺组织细胞来源的外泌体进行检测分析，旨在实现肺癌的实时诊断，结合PCA判别肺癌细胞来源外泌体的敏感性和特异性分别能达到95.3%和97.3%（95%CI），证实了基于PCA的SERS无创检测方法可实现判别NSCLC组织细胞来源的外泌体，从而可能实现对肺癌的临床诊断。近年来关于microRNA和外泌体等在肺癌诊断方面的研究如火如荼，但与拉曼光谱技术结合诊断肺癌的研究很少，给我们提供了新的思路。

## 3 结语

作为一种非侵入性检测技术，拉曼光谱检测技术诊断早期肺癌具有操作简单、快速有效、灵敏度及特异度高的优点。拉曼光谱检测技术的发展日新月异，结合不同组织细胞或体液样本的研究，可实现对肺癌的微创甚至无创检测，在临床早期肺癌诊断方面有着广阔的临床应用前景。研究开发稳定性及重现性更好、灵敏度及特异度更高的拉曼光谱检测技术，降低样本背景干扰对样本拉曼信号的影响，结合不同拉曼光谱数据的统计处理方法，进而实现肺癌的微创甚至无创的高效检测是今后科研工作者需要努力的方向。相信随着相关研究的深入发展，拉曼光谱检测技术在临床肺癌早期诊断中必将发挥更加重要的作用。
